# Colonic Polyp Study: Differences in Adenoma Characteristics Based on Colonoscopy History over 5-Year Follow-Up Period

**DOI:** 10.3390/jcm14010194

**Published:** 2024-12-31

**Authors:** Sang Hyun Park, Kwang Il Hong, Hyun Chul Park, Young Sun Kim, Gene Hyun Bok, Kyung Ho Kim, Dong Suk Shin, Jae Yong Han, Young Kwan Kim, Yeun Jong Choi, Soo Hoon Eun, Byung Hoon Lim, Kyeong Kun Kwack, The Korean Society of Digestive Endoscopy Polyp Study Workgroup

**Affiliations:** 1 Seoul SOK Clinic, Seoul 02566, Republic of Korea; technature2010@gmail.com; 2 Hiqhong IM Clinic, Incheon 22810, Republic of Korea; kihong111@hanmail.net; 3 Incheon SOK Clinic, Incheon 21573, Republic of Korea; giscope@hanmail.net; 4 Jangbaro Clinic, Uijeongbu 11815, Republic of Korea; genehyun@naver.com; 5 Sundu United Medical Clinic, Icheon 17420, Republic of Korea; minsoksumin@naver.com; 6 Samsungtop Internal Medicine, Bucheon 14537, Republic of Korea; lemonshake@hanmail.net; 7 Department of Internal Medicine, Seoul Bon Clinic, Seoul 04032, Republic of Korea; mdkoji@daum.net; 8 Dr. Kim Young Kwan’s Office, Seoul 04974, Republic of Korea; kimyk1017@empas.com; 9 Yonsei Choisun Internal Medicine Clinic, Incheon 21995, Republic of Korea; drcyj69@naver.com; 10 Hunhunhan Internal Medicine Clinic, Seoul 05351, Republic of Korea; dresh@paran.com; 11 Lim’s Internal Medicine Clinic, Gapyeong 12418, Republic of Korea; hoonee09@hanmail.net; 12 Seoul Medical Clinic, Seoul 02037, Republic of Korea; drmedi66@naver.com

**Keywords:** adenoma, colonoscopy, neoplasms, primary health care

## Abstract

**Background**: Timely detection and removal of colonic adenomas are critical for preventing colorectal cancer. **Methods**: This study analyzed differences in colonic adenoma characteristics based on colonoscopy history by reviewing the medical records of 14,029 patients who underwent colonoscopy between January and June 2020 across 40 primary medical institutions in Korea. **Results**: Adenoma and advanced neoplasia characteristics varied significantly with colonoscopy history (*p* < 0.05). In the first-time colonoscopy group, adenomas were more frequent in the sigmoid colon (S-colon) and rectum, with Is features and non-granular laterally spreading tumors. Advanced neoplasia was also more common in the S-colon and rectum, with Is and advanced-type features. In the <5-year group, adenomas were predominantly found in the transverse colon (T-colon) and descending colon (D-colon), with IIa and IIb features. Advanced neoplasia in this group was more frequent in the cecum and T-colon, with IIa and IIb features and laterally spreading tumors. In the ≥5-year group, adenomas were more commonly located in the ascending colon (A-colon) and cecum, with Ip features, while advanced neoplasia was more frequent in the A-colon and D-colon, also with Ip features. **Conclusions**: Although every segment of the colorectum should be carefully observed regardless of colonoscopy history, these findings suggest that prioritizing specific colonic segments for examination based on colonoscopy history may improve adenoma detection rates and reduce the incidence of colorectal cancer. However, further large-scale, prospective studies are needed to confirm these findings and support their application in clinical practice.

## 1. Introduction

Colon cancer is a leading cause of cancer-related mortality worldwide [1]. According to the adenoma–carcinoma sequence theory, most colon cancers develop from pre-existing adenomas, making their detection and removal critical for reducing both incidence and mortality [2]. Colonoscopy remains the primary tool for detecting colon cancer and monitoring patients after colonic adenoma resection. While guidelines for follow-up after colonoscopy and polypectomy are continuously updated, their applicability to routine clinical practice remains limited due to the scarcity of studies conducted in primary medical institutions [3,4,5,6]. Additionally, in real-world settings, colonoscopy is often performed more frequently than guideline recommendations, making it essential to identify adenoma characteristics and locations based on colonoscopy history to optimize patient management.

Previous studies on colonic adenomas have primarily focused on the incidence of adenomas or advanced adenomas based on the follow-up duration after colonoscopy. However, to our knowledge, no study has examined the predominant colonic locations of adenomas at different colonoscopy intervals. Therefore, this study aimed to evaluate the prevalence, differences in location, and characteristics of colonic adenomas in relation to colonoscopy history in primary medical institutions.

## 2. Materials and Methods

### 2.1. Study Population

This retrospective, cross-sectional, multicenter study analyzed the medical records of 14,056 patients aged 20–89 years who underwent colonoscopy for various indications, including screening, colonic symptoms, a history of colonic polyps, or a family history of colorectal cancer, between January and June 2020. The study followed the Polyp Study Protocol of the Korean Society of Digestive Endoscopy and was conducted across 40 primary medical institutions in Korea. The study findings have been reported in compliance with the STROBE checklist [7].

The exclusion criteria were age <20 years or ≥90 years; a history of colon cancer or inflammatory bowel disease; a withdrawal time of less than 6 min during colonoscopy; and an incomplete examination attributed to poor bowel preparation or difficulty in cecal intubation.

### 2.2. Chart Preparation

Patient demographics, including sex, age, weight, height, body mass index, diabetes status, and colonoscopy history (categorized as first time, within 5 years, or ≥5 years), were documented. The indications for colonoscopy were recorded for each patient: asymptomatic; abdominal pain, bloody stool, mucoid stool, diarrhea, constipation, abdominal discomfort (e.g., gas, indigestion, etc.), stool caliber changes, anemia, or weight loss; positive fecal occult blood test (FOBT); a history of colonic polyps; and a family history of colorectal cancer (in parents and siblings). Multiple indications were permitted for patients presenting with more than one symptom or risk factor.

### 2.3. Colonic Adenoma Analysis and Definitions

The total number, size, location, macroscopic shape (based on the Paris classification), and pathological findings of colonic adenomas detected during colonoscopy were analyzed. According to the US Multi-Society Task Force guidelines, an advanced adenoma was defined as an adenoma ≥1 cm, with (tubulo) villous histology, or with high-grade dysplasia. Advanced neoplasia was defined as either advanced adenoma or carcinoma [6].

### 2.4. Statistical Analysis

Values are represented as the mean ± standard deviation for continuous variables and the number and percentage for categorical variables. Patients were categorized into two age groups based on the recommended age for colonoscopy (50 years) and further divided into three colonoscopy history groups: first-time colonoscopy, <5 years, and ≥5 years since the last colonoscopy. A comparison of colonic adenoma characteristics among the groups was conducted using Pearson’s chi-squared test. Statistical analyses were performed using SPSS version 26 (IBM Corp, Armonk, NY, USA). All *p*-values were two-sided, with significance defined as *p* < 0.05.

## 3. Results

### 3.1. Patient Characteristics

A total of 27 patients with incomplete medical records were excluded, leaving 14,029 patients included in this study. Of these, 9986 patients (71.2%) were aged ≥50 years. The cohort consisted of 6949 men (49.5%; mean age: 55.9 ± 13.4 years) and 7080 women (50.5%; mean age: 57.2 ± 12.7 years). Among the 14,029 patients, 12.1% had diabetes, 40.6% were asymptomatic, 32.4% had a history of colonic polyps, 4.7% had a positive FOBT, and 3.0% had a first- or second-degree relative with colorectal cancer. Colonoscopy was performed for the first time in 26.8% of patients, within <5 years in 51.3%, and after ≥5 years in 17.4%.

### 3.2. Colonoscopy Indication

**Figure 1:**
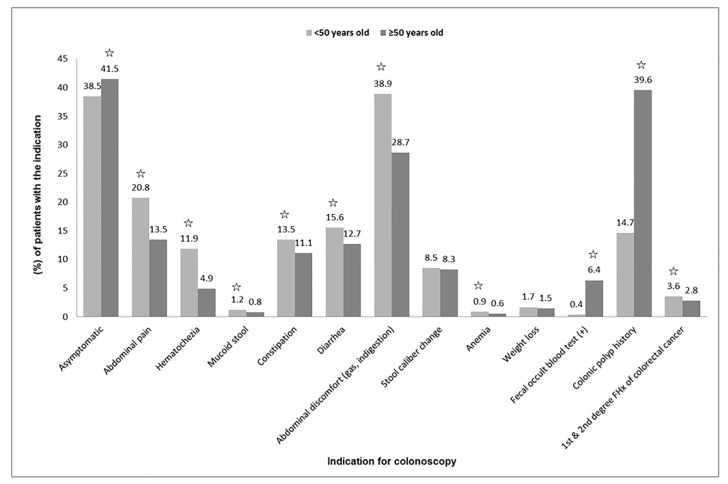
The comparison of colonoscopy indications between patients aged <50 years and those aged ≥50 years. Significant differences between the two groups are marked with a star (☆) (*p* < 0.05). FHx, family history.

The indications for colonoscopy are illustrated in Figure 1. Asymptomatic status, a positive FOBT, and polyp surveillance were significantly more frequent among patients aged ≥50 years. Conversely, all other indications, except changes in stool caliber and weight loss, were significantly more common in patients aged <50 years (*p* < 0.05).

### 3.3. Prevalence of Colonic Polyps

Colonic polyps, adenomas, advanced adenomas, advanced neoplasia, and carcinomas were identified in 8404 (59.9%), 5455 (38.9%), 822 (5.9%), 868 (6.2%), and 46 (0.3%) patients, respectively. The prevalence of polyps (65.2% vs. 46.8%), adenomas (45.3% vs. 23.2%), advanced adenomas (6.7% vs. 3.7%), and advanced neoplasia (7.1% vs. 4.0%) was significantly higher in patients aged ≥50 years compared to those aged <50 years (all *p* < 0.05). However, no significant difference was observed in the incidence of carcinomas between the two age groups (0.3% vs. 0.2%; *p* > 0.05).

### 3.4. Adenoma Characteristics According to Age

Next, a total of 10,217 adenomas detected in 14,029 patients were analyzed. Adenoma characteristics differed significantly between the two age groups (*p* < 0.05). Adenomas were more frequently observed in the sigmoid colon (S-colon) among patients aged <50 years, while they were more common in the ascending colon (A-colon) among those aged ≥50 years. Patients aged <50 years had a higher proportion of adenomas ≥1 cm in size (14.1% vs. 9%) but a lower frequency of IIa adenomas, as classified by the Paris classification (25.6% vs. 34.8%), compared to those aged ≥50 years. Serrated adenoma and high-grade dysplasia were more prevalent in the <50-year-old group, whereas villous adenoma and carcinoma in situ were more frequently observed in the ≥50-year-old group (Table 1).

### 3.5. Adenoma Characteristics According to Colonoscopy History

A total of 9740 adenomas from 13,396 patients with documented colonoscopy histories were analyzed. Adenomas were detected in 2683 (27.5%) cases in the first-time group, 5160 (53.0%) in the <5-year group, and 1897 (19.5%) in the ≥5-year group. Significant differences in adenoma characteristics were observed among the groups (*p* < 0.05). Adenomas were more commonly located in the transverse colon (T-colon) and descending colon (D-colon) in the <5-year group, the cecum and A-colon in the ≥5-year group, and the S-colon and rectum in the first-time group. Regarding morphology, type IIa and IIb adenomas were more frequent in the <5-year group, Ip adenomas in the ≥5-year group, and Is and non-granular laterally spreading tumors in the first-time group (Table 2).

### 3.6. Advanced Neoplasia Characteristics According to Colonoscopy History

Among the 9740 adenomas, 1008 were classified as advanced neoplasia. The male-to-female ratio for advanced neoplasia was 1.94:1. Most cases (88.3%) were reported in patients aged ≥50 years, with the earliest case detected at age 30. Advanced neoplasia was most frequently located in the S-colon, followed by the T-colon and A-colon. By colonoscopy history, advanced neoplasia was observed in 427 (42.4%) cases in the first-time group, 328 (32.5%) in the <5-year group, and 253 (25.1%) in the ≥5-year group.

Characteristics of advanced neoplasia, excluding size, differed significantly among the three groups (*p* < 0.05). In the first-time group, advanced neoplasia was more commonly observed in the S-colon and rectum, with Is and advanced-type features. In the <5-year group, it was more frequently located in the cecum and T-colon, characterized by IIa, IIb, and laterally spreading tumor features. In the ≥5-year group, advanced neoplasia was more common in the A-colon and D-colon, with Ip features (Table 3).

## 4. Discussion

Timely detection and removal of colonic adenomas are crucial for preventing colorectal cancer. This study aimed to improve adenoma detection methods by analyzing differences in adenoma characteristics based on colonoscopy history. The findings suggest that the right side of the colon warrants closer examination as the interval since the previous colonoscopy increases.

In this study, 51.3% of all patients underwent colonoscopy re-screening within 5 years of their initial screening, indicating a significant deviation from recommended guidelines in Korean primary medical institutions. Similar patterns have been reported in North America and Europe [8]. The decision to perform colonoscopy is often made without consideration of previous colonoscopy findings, leading to more frequent procedures. This practice is driven by concerns about missing potential neoplasms and doubts regarding the quality and accuracy of the prior colonoscopy results [9].

Colorectal cancer screening guidelines recommend initiating colonoscopy at the age of 50. In this study, the indications for colonoscopy varied by age group. Among patients aged ≥50 years, a positive FOBT result and a history of colonic polyps were more common reasons for colonoscopy, whereas symptom presence was the main indication for patients aged <50 years. However, 38.5% of patients aged <50 years were asymptomatic, a proportion higher than previously reported by Song et al. (32.7%) [10] and Hong et al. (30.6%) [11], suggesting an increased interest in colonoscopy among young populations.

In this study, adenomas were more frequently detected in the right colon than in the left colon (61.9% vs. 38.1%), which contrasts with previous studies reporting a higher frequency of left-sided adenomas (60.2–65.4%) compared to right-sided adenomas [12,13]. Our results suggest a potential shift in the prevalence of right-sided colonic adenomas, emphasizing the need for a more thorough examination of the right colon. This observation aligns with a recent study indicating an increased tendency for adenomas to be located in the proximal colon [14]. Additionally, we observed that adenoma characteristics varied significantly by age group. While the careful examination of the entire colorectum is essential regardless of age, patients aged <50 years require focused observations of the S-colon and T-colon, with particular attention to serrated adenomas. For patients aged ≥50 years, efforts should focus on identifying flat lesions in the A-colon and T-colon. These findings, consistent with prior research, underscore the importance of a comprehensive examination of the right colon, particularly in older patients [11,15].

In this study, the most common site for adenomas was the T-colon, followed by the A-colon and S-colon, regardless of colonoscopy history. However, adenoma characteristics varied significantly based on colonoscopy history. For instance, adenomas were more frequently observed in the T-colon and D-colon in the <5-year group, in the cecum and A-colon in the ≥5-year group, and in the S-colon and rectum in the first-time group. This pattern suggests that adenomas were initially more concentrated in the left colon during the first colonoscopy but became more widely distributed in the right colon as the interval from the prior colonoscopy increased. The higher miss rates of right-sided adenomas during colonoscopy may explain this finding. In a study by Kim et al. [16], the miss rates for total adenomas, right colonic adenomas, and left colonic adenomas were reported as 24.1%, 26.8%, and 21.4%, respectively. This implies that right-sided polyps missed during the first colonoscopy may become detectable over time as they grow larger or more prominent. Interestingly, our results indicated that adenomas were most frequently detected in the T-colon, regardless of the colonoscopy history. The T-colon, often considered easily observable, may in fact represent an unexpected blind spot, requiring a more thorough examination. Kashiwagi et al. [17] reported that polyp detection rates in the T-colon and S-colon significantly increased with longer withdrawal times during colonoscopy. This finding suggests that both the T-colon and S-colon may have lower observation times due to their anatomical complexity, leading to higher adenoma miss rates. Consequently, it is essential to carefully examine the entire colonic segment during colonoscopy, prioritize areas based on colonoscopy history, and allocate additional times to inspecting the right colon when longer intervals have elapsed since the previous colonoscopy.

In this study, the most common site of advanced neoplasia in the previous colonoscopy group was in the T-colon, followed by the A-colon or S-colon, whereas in the first-time group, it was the S-colon, followed by A-colon. Advanced neoplasia exhibited significant differences in characteristics according to the colonoscopy history, except for size. In the first-time group, advanced neoplasia was more frequent in the S-colon and rectum, characterized by Is and advanced-type features, including carcinoma in situ and high-grade dysplasia pathology. In the <5-year group, it was more prevalent in the cecum and T-colon, with IIa, IIb, and laterally spreading tumor features. In the ≥5-year group, advanced neoplasia was more common in the A-colon and D-colon. Although every segment of the colorectum should be carefully observed regardless of colonoscopy history, our findings suggest that the targeted examination of specific colonic segments, guided by colonoscopy history, may enhance the detection of adenomas and advanced neoplasia. These observations should be considered as preliminary and validated through future large-scale, prospective studies before definitive clinical recommendations can be made.

In this study, we were able to overcome selection bias, which was a shortcoming of previous studies involving single or tertiary medical institutions. We analyzed diverse groups with varying socioeconomic characteristics, enhancing the generalizability of our findings. However, the retrospective design of this study limited the availability of detailed patient information. Additionally, there is potential controversy regarding the precise localization of colonic segments during colonoscopy. According to a study by Manigrasso M et al. [18], the accuracy of colonoscopic lesion localization was 74.6%, dropping to 53.1% in the descending colon. Therefore, the detection rate or number of colonic polyps reported in specific segments may have been underestimated or overestimated in this study. Despite this limitation, our findings are novel and important. This large-scale, multicenter study provides valuable insights into colonoscopy practices and the prevalence of colonic adenomas in primary medical institutions across the country. Furthermore, we identified differences in adenoma characteristics over varying follow-up periods, offering practical suggestions for improving the current medical practice.

In conclusion, our findings provide valuable guidance for clinicians in prioritizing specific colonic segments for examination based on colonoscopy history and patient age. This approach may improve adenoma detection rates, facilitate early intervention, and reduce the incidence of colorectal cancer. Further large-scale, prospective studies in primary medical settings are needed to validate these results and support their application in real-world clinical practice.

## Figures and Tables

**Table 1:** Adenoma characteristics according to age.

Variables	Total Number of Cases (%) (*n* = 10,217)	Number of Patients Aged <50 Years (%) (*n* = 1452)	Number of Patients Aged ≥50 Years (%) (*n* = 8765)	χ^2^	The Critical Value Corresponding to Degrees of Freedom	*p*-Value
Location						
Cecum Ascending colon Transverse colon Descending colon Sigmoid colon Rectum	568 (5.5) 2480 (24.3) 3283 (32.1) 1293 (12.7) 2085 (20.4) 508 (5.0)	84 (5.8) 295 (20.3) 436 (30.0) 197 (13.6) 362 (24.9) 78 (5.4)	484 (5.5) 2185 (24.9) 2847 (32.5) 1096 (12.5) 1723 (19.7) 430 (4.9)	32.04	11.070	0.01 *
Size						
<0.5 cm 0.5–<1 cm 1–<2 cm ≥2 cm	3431 (33.6) 5793 (56.7) 899 (8.8) 94 (0.9)	427 (29.4) 821 (56.5) 182 (12.5) 22 (1.6)	3004 (34.3) 4972 (56.7) 717 (8.2) 72 (0.8)	42.14	7.815	0.01 *
Shape						
Ip Is IIa IIb IIc III LST-G LST-NG	973 (9.5) 5432 (53.2) 3470 (34.0) 255 (2.6) 4 (0.0) 5 (0.0) 33 (0.3) 45 (0.4)	115 (7.9) 922 (63.5) 372 (25.6) 31 (2.2) 0 (0.0) 1 (0.1) 2 (0.1) 9 (0.6)	858 (9.8) 4510 (51.5) 3048 (34.8) 274 (3.1) 4 (0.0) 4 (0.0) 31 (0.4) 36 (0.4)	77.22	11.070	0.01 *
Pathology						
TA TVA VA SA CIS HGD	9773 (95.6) 130 (1.3) 6 (0.1) 258 (2.5) 6 (0.1) 44 (0.4)	1348 (92.8) 12 (0.8) 0 (0.0) 80 (5.6) 0 (0.0) 12 (0.8)	8425 (96.1) 118 (1.4) 6 (0.1) 178 (2.0) 6 (0.1) 32 (0.3)	68.32	9.488	0.01 *

* Significant *p*-values are indicated in bold (*p* < 0.05). CIS, carcinoma in situ; HGD, high-grade dysplasia; LST-G, laterally spreading tumor, granular; LST-NG, laterally spreading tumor, non-granular; SA, serrated adenoma; TA, tubular adenoma; TVA, tubulovillous adenoma; VA, villous adenoma.

**Table 2:** The characteristics of adenomas based on the follow-up period since the last colonoscopy.

Variables	Number of Adenomas (%) at Previous CFS Within 5 Years (*n* = 5160)	Number of Adenomas (%) at Previous CFS at ≥5 Years (*n* = 1897)	Number of Adenomas (%) During First-Time CFS (*n* = 2683)	χ^2^	The Critical Value Corresponding to Degrees of Freedom	*p*-Value
Location						
Cecum Ascending colon Transverse colon Descending colon Sigmoid colon Rectum	287 (5.6) 1227 (23.8) 1782 (34.5) 687 (13.3) 964 (18.7) 213 (4.1)	114 (6.0) 483 (25.5) 619 (32.6) 209 (11.0) 371 (19.6) 101 (5.3)	129 (4.8) 658 (24.5) 751 (28.0) 342 (12.7) 649 (24.2) 154 (5.8)	68.93	18.307	0.01 *
Size						
<0.5 cm 0.5–<1 cm 1–<2 cm ≥2 cm	2007 (39.0) 2809 (54.4) 312 (6.0) 32 (0.6)	584 (30.7) 1128 (59.5) 168 (8.9) 17 (0.9)	661 (24.6) 1618 (60.4) 366 (13.6) 38 (1.4)	255.19	12.592	0.01 *
Shape						
Ip Is IIa IIb IIc III LST-G LST-NG	447 (8.7) 2474 (47.9) 2058 (39.9) 147 (2.8) 2 (0.1) 3 (0.1) 12 (0.2) 17 (0.3)	195 (10.3) 951 (50.1) 685 (36.1) 49 (2.6) 0 (0.0) 0 (0.0) 7 (0.4) 10 (0.5)	269 (10.0) 1644 (61.3) 687 (25.6) 52 (1.9) 2 (0.1) 1 (0.0) 10 (0.4) 18 (0.7)	184.97	18.307	0.01 *
Pathology						
TA TVA VA SA CIS HGD	4995 (96.7) 28 (0.5) 1 (0.1) 123 (2.4) 1 (0.1) 12 (0.2)	1810 (95.3) 34 (1.8) 3 (0.2) 43 (2.3) 1 (0.1) 6 (0.3)	2518 (93.8) 62 (2.3) 2 (0.1) 74 (2.8) 4 (0.1) 23 (0.9)	92.89	15.507	0.01 *

* Significant *p*-values are indicated in bold (*p* < 0.05). CFS, colonofiberoscopy; CIS, carcinoma in situ; HGD, high-grade dysplasia; LST-G, laterally spreading tumor, granular; LST-NG, laterally spreading tumor, non-granular; SA, serrated adenoma; TA, tubular adenoma; TVA, tubulovillous adenoma; VA, villous adenoma.

**Table 3:** The characteristics of advanced neoplasia based on the follow-up period since the last colonoscopy.

Variables	Total Number of Advanced Neoplasia Cases (%) (*n* = 1008)	Number of Advanced Neoplasia Cases (%) at Previous CFS Within 5 Years (*n* = 328)	Number of Advanced Neoplasia Cases (%) at Previous CFS at ≥5 Years (*n* = 253)	Number of Advanced Neoplasia Cases (%) During First-Time CFS (*n* = 427)	χ^2^	The Critical Value Corresponding to Degrees of Freedom	*p*-Value
Sex							
Male Female	665 (66.0) 343 (34.0)	212 (64.6) 116 (35.4)	168 (66.4) 85 (33.6)	285 (66.7) 142 (33.3)	0.39	5.991	0.82
Location							
Cecum A-colon T-colon D-colon S-colon Rectum	51 (5.1) 222 (22.0) 229 (22.7) 130 (12.9) 257 (25.5) 119 (11.8)	21 (6.4) 69 (21.0) 98 (30.0) 43 (13.1) 71 (21.6) 26 (7.9)	10 (4.0) 60 (23.7) 67 (26.4) 38 (15.0) 51 (20.2) 27 (10.7)	20 (4.7) 93 (21.7) 64 (15.0) 49 (11.5) 135 (31.6) 66 (15.5)	44.44	18.307	0.01 *
Size							
<0.5 cm 0.5–<1 cm 1–<2 cm ≥2 cm	13 (1.3) 58 (5.8) 837 (83.0) 100 (9.9)	3 (0.9) 14 (4.3) 284 (86.6) 27 (8.2)	5 (2.0) 19 (7.5) 205 (81.0) 24 (9.5)	5 (1.2) 25 (5.9) 348 (81.4) 49 (11.5)	6.72	12.592	0.347
Shape							
Ip Is IIa IIb IIc III LST-G LST-NG Advanced	193 (19.1) 579 (57.5) 94 (9.3) 72 (7.1) 1 (0.1) 3 (0.3) 22 (2.2) 27 (2.7) 17 (1.7)	61 (18.6) 171 (52.1) 38 (11.6) 34 (10.4) 0 (0) 1 (0.3) 10 (3.0) 11 (3.4) 2 (0.6)	51 (20.1) 150 (59.3) 18 (7.1) 22 (8.7) 0 (0) 1 (0.4) 4 (1.6) 4 (1.6) 3 (1.2)	81 (19.0) 258 (60.5) 38 (8.9) 16 (3.7) 1 (0.2) 1 (0.2) 8 (1.9) 12 (2.8) 12 (2.8)	29.12	26.296	0.02 *
Pathology							
TA TVA VA CIS HGD Carcinoma	776 (77.0) 130 (12.8) 6 (0.6) 6 (0.6) 44 (4.4) 46 (4.6)	280 (85.4) 28 (8.5) 1 (0.3) 1 (0.3) 12 (3.7) 6 (1.8)	191 (75.4) 40 (15.8) 3 (1.2) 1 (0.4) 9 (3.6) 9 (3.6)	305 (71.4) 62 (14.5) 2 (0.5) 4 (0.9) 23 (5.4) 31 (7.3)	30.19	18.307	0.01 *

* Significant *p*-values are indicated in bold (*p* < 0.05). A-colon, ascending colon; CFS, colonofiberoscopy; CIS, carcinoma in situ; D-colon, descending colon; HGD, high-grade dysplasia; LST-G, laterally spreading tumor, granular; LST-NG, laterally spreading tumor, non-granular; S-colon, sigmoid colon; SA, serrated adenoma; TA, tubular adenoma; T-colon, transverse colon; TVA, tubulovillous adenoma; VA, villous adenoma.

## Data Availability

Data are available from the corresponding author on reasonable request.
